# Sexual violence among adolescent girls and young women in Malawi: a cluster-randomized controlled implementation trial of empowerment self-defense training

**DOI:** 10.1186/s12889-018-6220-0

**Published:** 2018-12-04

**Authors:** Michele R. Decker, Shannon N. Wood, Esther Ndinda, Gayane Yenokyan, Jacob Sinclair, Nankali Maksud, Brendan Ross, Benjamin Omondi, Martin Ndirangu

**Affiliations:** 1 Department of Population, Family & Reproductive Health Director, Women’s Health & Rights Program, Johns Hopkins Bloomberg School of Public Health, Baltimore, MD USA; 2 Center for Public Health & Human Rights, Johns Hopkins Bloomberg School of Public Health, 615 N. Wolfe Street, E4142, Baltimore, MD 21205 USA; 3 Ujamaa-Pamodzi, Lilongwe, Malawi; 4 Johns Hopkins Biostatistics Center, Johns Hopkins Bloomberg School of Public Health, Baltimore, MD USA; 5 Ujamaa-Africa, Nairobi, Kenya; 6 UNICEF, Lilongwe, Malawi; 7 UNICEF, Mogadishu, Somalia

## Abstract

**Background:**

Globally, sexual violence is prevalent, particularly for adolescent women. This cluster-randomized controlled implementation trial examines empowerment self-defense (ESD) for sexual assault risk reduction among school-age women in Malawi.

**Methods:**

The unit of randomization and analysis was the school (*n* = 141). Intervention participants received a 12-h intervention over 6 weeks, with refreshers. Primary outcomes were past-year prevalence and incident rate of sexual violence. Secondary outcomes included confidence, self-defense knowledge, and, for those victimized, violence disclosure. Interaction effects on outcomes were evaluated with Poisson models with school-correlated robust variance estimates for risk ratios and incident rate ratios (baseline *n* = 6644, follow-up *n* = 4278).

**Results:**

Past-year sexual assault prevalence was reduced among intervention students (risk ratio [RR] 0.68, 95% CI 0.56, 0.82), but not control students (interaction effect *p* < 0.001). Significant increases in self-defense knowledge were observed solely among intervention students (RR 3.33, 95% CI 2.76, 4.02; interaction effect *p* < 0.001). Significant changes in sexual violence prevalence and knowledge were observed for both primary and secondary students. Favorable reductions were also observed in sexual violence incident rate among students overall (interaction effect *p* = 0.01).

**Conclusions:**

This intervention reduced sexual violence victimization in both primary and secondary school settings. Results support the effectiveness of ESD to address sexual violence, and approach the elimination of violence against women and girls set forth with Sustainable Development Goal #5. Implementation within the education system can enable sustainability and reach.

**Trial registration:**

Pan African Clinical Trials Registry PACTR201702002028911. Registered 09 February 2017. Retrospectively registered.

## Background

Gender-based violence (GBV), i.e., violence perpetrated based on sex or gender identity [1], has been recognized as a public health and human rights issue since 1993 [2]. Globally, an estimated one in three women experiences physical or sexual violence [3]. Physical, sexual and mental health morbidities resulting from gender-based violence are well-characterized [4, 5] and include unintended pregnancy and sexually transmitted infection including HIV. Adolescents are at high risk for GBV [6]; accordingly, research priorities for adolescent sexual and reproductive health in low and middle-income countries (LMICs) highlight GBV prevention [7]. Adolescents’ young age and relative inexperience can constrain their relationship power and incur risk, particularly with older partners [8–11]. Violence can set young women on a trajectory for future violence [12, 13] and sexual risk behavior [14].

Malawi is a critical setting for preventing and responding to GBV, particularly sexual violence against young women, for whom the prevention evidence lags behind. Recent national data from Malawi reveal that an estimated one in five (21.8%) young adult women experienced sexual abuse prior to age 18, primarily perpetrated by boyfriends, classmates and acquaintances [15]. Other nationally representative data estimate that one in four (25%) sexually experienced women ages 15–19 characterize their sexual debut as forced, well above estimates pooled across all available data globally (15%) and within the region (21%) [6]. For youth, gender-based power disparities can be exacerbated by age-based power disparities; for almost half of female childhood (< 18 years) sexual abuse victims, perpetrators were five or more years older [15]. The health and development impact of sexual violence is profound. As in other settings, sexual violence against young women in Malawi is associated with poor outcomes across domains of sexual and reproductive health [16], and mental health [16]. Victimization can also undermine school enrollment and progress [17], thus compromising women’s future engagement in political, business and economic sectors.

These patterns play out against pervasive gender-based violence and stark gender disparities within the nation. An estimated 40% of ever-married women in Malawi have experienced intimate partner violence in their lifetimes, with past-year abuse affecting 30% [18]. National data also confirm the dominance of husbands in decision-making over household spending and access to health care [18]. The 2014 Gender Inequality Index, which reflects gender inequalities in reproductive health, empowerment and economic activity, ranked Malawi at 140 out of 154 countries, largely reflecting maternal mortality and adolescent fertility indicators as well as gender-based educational disparities [19]. These structural-level factors and adolescents’ experiences of sexual violence create an entrenched, mutually reinforcing cycle whereby tolerance of abuse, and structural gender inequalities perpetuate sexual violence. Simultaneously, sustained violence undermines gender equality through conveying the notion that young women are not valued, and by curtailing their engagement in education, employment and mobility based on safety concerns [20, 21].

Addressing sexual violence in Malawi and other endemic settings requires responding to structural forces, including social norms that tolerate sexual violence and expect silence and isolation in response, particularly for young women. Empowerment self-defense (ESD) is an interactive training experience that prepares participants for mental, verbal and physical self-defense through bolstering verbal and physical safety skills, and imparts the self-confidence to implement them. Safety promotion can reduce danger, and shift power away from potential abusers. Moreover, through preparation and practice, participants cultivate use of their voice and personal power in a striking contrast to social expectations of silence and compliance that enable continued violence perpetration with impunity. ESD provides active tools for resistance, thus responding to structural forces that disempower young women, blame them for victimization and undermine safety.

While a comprehensive approach to sexual violence prevention necessarily entails addressing perpetration, there remains a sustained need to assist women in resisting sexual violence, particularly in high-prevalence contexts such as Malawi. As leading scholars articulate, such risk reduction interventions can and must be implemented in a way that does not blame victims for their experiences,  but rather strengthens resistance to victim-blaming for such experiences [22]. Recent reviews suggest ESD is a promising yet underutilized strategy for violence risk reduction, though discourse and evaluation to date has primarily focused on college and university campuses, predominantly in higher income contexts [22, 23]. In college and university settings, sexual violence interventions that incorporate self-defense have been shown to reduce sexual assault [24] including both completed and attempted rape [25]. These reductions in violence may reflect the increases observed in both confidence in resisting sexual violence [24, 26] and behavioral strategies for self-protection [26]. ESD has had limited uptake in LMIC settings; one notable exception is its implementation and evaluation in the densely populated urban communities of Nairobi, Kenya, where it was found to reduce sexual assault incidence among young women [27, 28], and also buffered against pregnancy-related school dropout, suggesting a cascade impact on health and well-being [29].

This study sought to determine the effect of a standardized 6-week ESD program (IMPower) on sexual violence outcomes among primary and secondary school girls in three distinct districts of Malawi, relative to a control condition who received life-skills training. We also explore impact on proximal outcomes including self-defense knowledge, and confidence/ self-efficacy, and disclosure of violence. This cluster-randomized implementation trial was conducted in the context of the expansion of IMpower into Malawi schools through the UNICEF Safe Schools Initiative. It extends the limited knowledge base on ESD in LMICs, and explores its value in more diffuse and heterogeneous settings.

## Methods

### Setting, recruitment and data collection

This study was implemented in the Malawi districts of Lilongwe, Dedza, and Salima, selected for heterogeneity and based on designation as priority, high-need settings for UNICEF’s Safe Schools Program. Lilongwe, the capital, is the most urbanized site. Dedza lies south of Lilongwe, in the mountains on the Mozambique border. Salima is located on the lake. Within districts, primary and secondary schools were selected for stratified randomization from a full listing of schools participating in UNICEF’s Safe Schools program by UNICEF’s field assessment team.

Within each selected school, research staff drew a simple random sample of students for activity participation (intervention or control) with the goal of a 20:1 student to instructor ratio. Based on instructor capacity in Lilongwe and Dedza, approximately 60 female students were selected for activity participation per school; in Salima where more staff were available, approximately 100 female students were selected per school. In most settings, participating classes included primary school classes 5, 6, 7, 8 and secondary school forms 1, 2, 3, 4. In Salima, Class 8 was not included in the selection pool based on retention concerns as many Class 8 students graduate. Sessions took place after school and were split as necessary for larger groups. Students within participating schools were blinded to intervention or control status. Within schools, simple random sampling was achieved by gathering participating school classes outside for circulation of an opaque plastic tin containing a pre-determined mix of beads specific to the size of each school. Students each selected a single bead; bead color red indicated random selection into the activities (intervention or control) underway at their school. In several schools, administrator concerns emerged regarding the use of red beads for random selection, and black instructor uniforms, which led to color modification for these procedures.

### Study population and retention

**Fig. 1:**
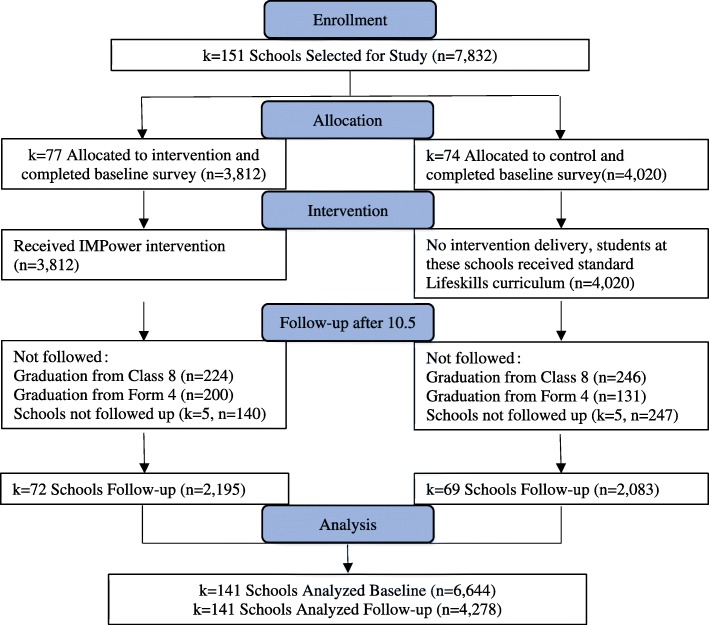
Participant flow chart

Baseline data was collected from February to June 2015 (See Fig. 1). Sample size was determined on the basis of implementation capacity. Follow-up data collection occurred from November 2015–May 2016. Follow-up data collection was not attempted in ten schools (5 Lilongwe, 4 Dedza, 1 Salima) based on the aforementioned administrator concerns; additionally students in Class 8 and Form 4 were not followed after completing school examinations; yielding an effective baseline sample of 6644 students. Follow-up data was obtained  for 3311 primary school students and 967 secondary school students (total *n* = 4278).

### IMPower intervention activities

IMPower consists of weekly, 2-h sessions for 6 weeks for a total of 12 h of interactive, empowerment self-defense training. Because physical interventions can escalate situations of potential violence, IMPower emphasizes early recognition of boundary testing, negotiation, diffusion and distraction tactics, and verbal assertiveness over physical self-defense, with the guidance that physical tactics should only be used if they are the last and best option. IMpower teaches boundary recognition and boundary setting (e.g., name harmful behaviors, warn about consequences), negotiation and diffusion tactics, verbal assertiveness (e.g., yell if threatened), and physical defense skills, with the self-efficacy to implement these skills. The physical skills comprise closed target skills, weapons and targets. After the six weeks, two-hour refresher courses are performed every 3–6 months. IMPower was developed by No Means No Worldwide (NMNW), a US-based NGO. An extensive formative phase was conducted to adapt IMPower for the Malawi context. Following community sensitization and structured discussions with key stakeholders, program adaptations included an emphasis on verbal and negotiation skills, with clarification that a physical response, including physical self-defense, is a last resort in a situation of danger. IMPower instructors are carefully selected with preference for experience with youth and on issues of GBV, and capacity for teaching and community organizing. All instructors attend a 3-week, 126-h intensive training in Lilongwe. After certification, instructors are deployed, most often to their home districts, for a 6-month period of co-teaching with an experienced instructor, followed by independent teaching.

### Control condition activities

Students randomized to the control condition received the standard of care for youth in schools, specifically the Lifeskills program. This standard 2-h Malawian school program covers adolescent health topics including puberty, menstruation, hygiene, sex education, STIs/HIV, and pregnancy prevention. These students also received two-hour refresher classes at 3–6 months also focused on puberty and hygiene at 10.5 months, prior to completing follow-up surveys.

### Supporting sexual violence survivors: The SASA program

IMPower participants who disclosed sexual violence during the program were referred to the Sexual Assault Survivors Anonymous (SASA) support program. This voluntary program offers weekly meetings with the goal of healing. From the IMPower program, 56 students in Lilongwe, 46 in Dedza, and 70 in Salima, were voluntarily linked to SASA support services.

### Randomization

The unit of randomization was the school, to minimize risk of contamination given the intensity of the IMPower intervention. Within district, primary and secondary schools were grouped into blocks based on approximate size, and randomized within block to intervention or control conditions using SPSS (allocation ratio 1:1). Participants were assigned to intervention or control condition based on school. For logistical purposes regarding planning program duration, school administrators were aware of assignment to intervention condition prior to study implementation. While every effort was made to blind students and teachers to intervention arm, intervention status could have been inadvertently been revealed to teachers or students.

### Data collection procedures

Informed consent followed UNICEF guidelines. Teacher consent was undergone during the formative phase to allow classroom participation of students. Written consent was obtained from parents and students prior to participation in the program and evaluation research.

Data collection procedures were designed to maximize confidentiality, in accordance with best practices for violence-related research [30]. No names or identifiers were collected. Participants self-administered baseline and follow-up surveys, aided by two instructors present in each class: one for reading the measures aloud and the other for monitoring the class to ensure confidentiality. Each question and answer choice was read aloud to the entire class in both English and Chichewa. Instructors from the local district were present to ensure that the district-specific dialect of Chichewa was used for survey administration, as terms vary by district. The students were given the opportunity to ask questions before selecting their own answer choice. To minimize deductive disclosure related to speed of survey completion, a response was required for each question, including “never had sex” or “never experienced sexual violence.” To maximize confidentiality, following survey completion, participants deposited their survey in a locked ballot box [31] to be taken directly to the research office, thus limiting the risk of instructors or data collectors inadvertently viewing responses. Students in both study arms had the option to self-refer to study staff and/or teachers for violence-related support and connection to local services.

### Measures

All measures were self-reported. They were adapted from prior evaluations of the IMPower program in Kenya [27, 28, 32] for comparability, and piloted in Malawian primary and secondary schools and with Chichewa-speaking instructor groups for feasibility and acceptability. Survey instruments were designed to maximize efficiency and feasibility for this demonstration study, as well as confidentiality. Primary outcomes were past year prevalence and past year incident rate of forced sex; for comparability with past evaluations. The forced sex outcome was assessed with a single item used for Kenya-based evaluations of IMPower [27, 28, 32], specifically, “Since you took the No Means No survey or in the past one year, have you ever been forced against your will to have sex (penetration of your vagina, anus or mouth with a man’s penis or another object)?” with a follow-up question “If so, how many times?”. School-level prevalence reflects the total number of participants who reported any forced sex over the referent period, divided by the total number of participants in the school. Incident rate was computed as mean number of forced sexual incidents per person-time within school, converted to adjust for time frame differences between the baseline (12 months) and follow-up period (10.5 months). Prevalence and disclosure outcomes are presented as proportions for interpretability; statistical inferences were robust in sensitivity analysis with time-adjusted estimates (data not shown).

Intermediate outcomes were confidence, knowledge, and sexual violence disclosure. Confidence measures were developed specifically for IMPower, specifically, “If I am attacked by a bigger man I feel confident that I can defend myself” and “Is it okay to use force and even injure anyone who is known to me if he is forcing me to have sex and will not listen to me (e.g., brother, boyfriend, father, cousin)?” Knowledge pertained to the self-defense skills taught, specifically: “If I am grabbed by an attacker what should I use to free myself?” and “The main aim of self-defense is to?” Both confidence and knowledge were then transformed into scores—if students answered both questions correctly, they were considered to have “complete” knowledge or “high” confidence, respectively, with those answering only one or zero serving as the referent group. Disclosure of sexual violence was measured among girls who had experienced violence in the past year/since the baseline survey from the question “Did you tell anyone about it?” and analyzed dichotomously. Participants with past-year forced sex were asked to indicate perpetrator(s), which were aggregated into the following groups: boyfriend, friend, relative (including stepfather/father and brother), neighbor, known adult (comprising teacher, pastor, police, doctor, and imam), stranger (including gangster), and other perpetrator. Formative research documented presence of Chinamwali, a Malawian rite of passage that can include forced sex, undergone by adolescent girls at the start of puberty. Chinamwali experiences were assessed via “In some communities in Malawi girls attend/undergo chinamwali. Have you gone through chinamwali?” These items were the only measures unique to Malawi.

### Analysis

Data were entered by a single individual and independently reviewed for accuracy by a second individual. The unit of analysis is the school level. We attempted anonymous matching of baseline and follow-up surveys for individual-level statistical analysis; participants self-created an identification code based on responses to questions for which only they would know the answer. The elements of the self-created identification codes proved suboptimal; for example, participants had limited knowledge regarding month and year of birth, and matching across time periods was poor. Individual-level baseline characteristics of participants by school type and intervention arm were compared using chi-squared statistics. Subsequent matching analyses and comparisons across time points were all done at the school-level given the suboptimal matching of individual-level data.

To support aggregate analysis at the school level, baseline and follow-up data points were collapsed at the school level to obtain means and counts. The primary and intermediate endpoints included past-year sexual violence prevalence, past-year sexual violence incident rate (primary), disclosure of abuse, self-defense knowledge, and confidence (intermediate). Poisson regression with cluster-correlated robust variance estimates was used to estimate either risk ratios or incident rate ratios per student-month comparing intervention arms by time point. The model included arm, time point (baseline vs. endline), and time-arm interaction to test within-arm baseline to endline relative change. School size (or a product of school size by time period) was used as an offset term in the Poisson regression to constrain the coefficient to one and use it as the denominator for the risk and rates. Results were subsequently stratified by type of school (primary/secondary) and district. Additionally, perpetrator groups were compared between baseline and follow-up; *p*-values were estimated using generalized linear models with binomial distribution and logit link and school-correlated variance, using each type of perpetrator as the outcome. The sample size fluctuates slightly to accommodate small amounts of missing data.  All statistical analyses were conducted in STATA 14 (STATCORP. College Station, TX).

All procedures were approved by the Malawi National Commission for Science and Technology (Ref. No. NCST/RTT/2/6). Johns Hopkins conducted post-hoc evaluation analysis with support from UNICEF, and received a non-human subject IRB determination given the anonymous nature of data. This trial was registered with Pan African Clinical Trials Registry (PACTR201702002028911) in February 2017; trial registry did not occur in advance of participant enrollment due to ambiguity on eligibility for trial registry given the applied (rather than clinical) nature of the study. Resource constraints prevented data collection on the pregnancy-related school dropouts initially proposed within the protocol as well as implementation of the companion programming for young men; thus the current analysis reports solely on sexual assault outcomes. The intervention promotes boundary setting through voice and action, with physical self-defense as a last resort. There are possible harms that could result from self-defense, including injury. No adverse events have been reported in past trials [27, 32] of this intervention. For this implementation trial, study staff were directed to immediately report any adverse events to the Ujamaa office; no adverse events were detected.

## Results

### Individual baseline characteristics

Table 1 disaggregates responses of the 5199 the primary school and 1445 secondary school students at baseline by intervention status and school type. Mean age of primary school students was 15.28 (SD = 2.06) and mean age of secondary school students was 19.55 (SD = 2.70). Salima district comprised approximately 50% of primary school students, whereas approximately 70% of secondary school students were from Lilongwe. Sixteen percent of primary school girls and 26% of secondary school girls had ever been forced to have sex. Prevalence of previous year forced sex was slightly lower than ever forced, with 10% of primary school girls and 18% of secondary school girls reporting forced sex victimization in the past year. Of girls who reported forced sex experiences at baseline, 49% of primary school girls reported only one incident within the past year, whereas 63% of secondary school girls reported multiple incidents.

**Table 1:** Baseline Characteristics by Intervention Group^§^

	Primary School			Secondary School		
	Intervention (*n* = 2491) n (%)	Control (*n* = 2708) n (%)	Overall (*n* = 5199) n (%)	Intervention (*n* = 757) n (%)	Control (*n* = 688) n (%)	Overall (*n* = 1445) n (%)
District						
Lilongwe	702 (28.2)	569 (21.0)	1271 (24.5)^*^	495 (65.4)	497 (72.2)	992 (68.7)^*^
Dedza	484 (19.4)	654 (24.2)	1138 (21.9)	50 (6.6)	59 (8.6)	109 (7.5)
Salima	1305 (52.4)	1485 (54.8)	2790 (53.7)	212 (28.0)	132 (19.2)	344 (23.8)
School Class						
Class 5	929 (37.7)	1123 (41.8)	2052 (39.8)^*^	–	–	–
Class 6	819 (33.2)	849 (31.6)	1668 (32.4)	–	–	–
Class 7	718 (29.1)	712 (26.5)	1430 (27.8)	–	–	–
Form 1	–	–	–	286 (37.8)	242 (35.5)	528 (36.7)
Form 2	–	–	–	225 (29.7)	204 (30.0)	429 (29.9)
Form 3	–	–	–	245 (32.4)	235 (34.5)	480 (33.4)
Chinamwali						
Underwent	721 (29.3)	1022 (38.0)	1743 (33.8)^*^	240 (32.5)	219 (32.1)	459 (32.3)
Did not undergo	1742 (70.7)	1667 (62.0)	3409 (66.2)	499 (67.5)	464 (67.9)	963 (67.7)
Ever forced to have sex						
Yes	407 (16.4)	403 (14.9)	810 (15.6)	220 (29.3)	154 (22.4)	374 (26.0)^*^
No	2082 (83.7)	2304 (85.1)	4386 (84.4)	531 (70.7)	534 (77.6)	1065 (74.0)
Past year forced sex						
None	2164 (90.3)	2375 (90.6)	4539 (90.4)	584 (80.9)	550 (82.8)	1134 (81.8)
1 time	120 (5.0)	112 (4.3)	232 (4.6)	53 (7.3)	41 (6.2)	94 (6.8)
2 times	47 (2.0)	56 (2.1)	103 (2.1)	35 (4.9)	43 (6.5)	78 (5.6)
3 times	**	**	**	23 (3.2)	15 (2.3)	38 (2.7)
4+ times	**	52 (2.0)	**	27 (3.7)	15 (2.3)	42 (3.0)

§ Sample size fluctuates slightly to accommodate small amounts of missing data, *χ^2^ test significant at the 0.05 level, **cell with < 2% suppressed

### Primary outcomes

Across all students, baseline past-year sexual violence was prevalent in both intervention (15.2%) and control arms (13.8%; Table 2). By follow-up, prevalence in the intervention arm dropped to 9.2%(Risk Ratio(RR)_Intervention_ 0.59 [95% CI 0.49–0.72]), while control arm prevalence remained approximately steady at 14.5% (RR_Control_1.04[0.86–1.26]); interaction effect *p*-value < 0.001. Overall incident rates followed a similar trend; they were comparable at baseline (19.6 per 1000 student-months vs. 19.3 control arm), decreased significantly by follow-up in the intervention arm (16.3; Rate Ratio_Intervention_ = 0.82 [0.67–1.00]), and increased in the control arm (24.0; Rate Ratio_Control_ = 1.22[0.95–1.57].The overall interaction effect favored the intervention arm (*p*-value =0.01).

**Table 2:** Primary and secondary outcomes across intervention and control arms and intervention effects on changes from baseline to follow-up

	Baseline			Follow-up			Difference Baseline to Follow-up				Intervention Effect
	Intervention	Control		Intervention	Control		Intervention		Control		
	% (95% CI) *(rate) (95% CI)*		*p*-value^a^	% (95% CI) *(rate) (95% CI)*		*p*-value^a^	RR (95% CI)	*p*-value^b^	RR (95% CI)	*p*-value^b^	*p*-value^c^
All Schools Primary outcomes											
Past year sexual violence prevalence	15.2% (12.8–18.1)	13.8% (11.6–16.3)	0.41	9.2% (7.7–11.0)	14.5% (12.0–17.6)	**0.001**	**0.59** **(0.49–0.72)**	**< 0.001**	1.04 (0.86–1.26)	0.70	**< 0.001**
School incident rate^d^	*(19.6)* *(16.1–23.9)*	*(19.3)* *(15.7–23.8)*	0.92	*(16.3)* *(13.3–20.0)*	*(24.0)* *(19.5–29.4)*	**0.01**	0.82 (0.67–1.00)	**0.05**	1.22 (0.95–1.57)	0.12	**0.01**
Intermediate outcomes											
High confidence	41.2% (35.7–47.6)	26.2% (22.8–30.2)	**< 0.001**	55.0% (50.1–60.3)	33.2% (28.9–38.2)	**< 0.001**	**1.33** **(1.17–1.50)**	**< 0.001**	**1.26** **(1.06–1.49)**	**0.01**	0.63
Correct knowledge	15.0% (12.0–18.8)	7.0% (5.3–9.2)	**< 0.001**	50.4% (45.6–55.7)	8.9% (6.8–11.8)	**< 0.001**	**3.33** **(2.76–4.02)**	**< 0.001**	1.27 (0.95–1.70)	0.11	**< 0.001**
Sexual violence disclosure^e^	73.0% (67.4–79.1)	68.2% (63.4–73.2)	0.21	83.7% (78.4–89.3)	74.8% (70.8–79.1)	**0.01**	**1.14** **(1.04–1.26)**	**0.007**	**1.10** **(1.01–1.19)**	**0.02**	0.50
Primary School											
Past year sexual violence prevalence	13.0% (10.5–16.0)	12.1% (10.5–14.1)	0.62	7.5% (6.1–9.3)	11.8% (9.9–14.1)	**0.001**	**0.58** **(0.45–0.74)**	**< 0.001**	0.97 (0.78–1.20)	0.78	**0.002**
School incident rate^d^	*(15.3)* *(12.3–19.0)*	*(16.9)* *(13.9–20.6)*	0.50	*(13.6)* *(10.7–17.4)*	*(20.2)* *(16.2–25.4)*	**0.02**	0.88 (0.71–1.11)	.29	1.20 (0.88–1.62)	0.25	0.12
High confidence	37.1% (31.4–43.8)	23.5% (19.9–27.8)	**< 0.001**	50.9% (45.5–57.0)	29.2% (25.1–34.1)	**< 0.001**	**1.37** **(1.18–1.59)**	**< 0.001**	1.24 (1.00–1.54)	0.05	0.46
Correct knowledge	11.6% (8.9–15.1)	5.3% (3.9–7.2)	**< 0.001**	45.7% (40.6–51.5)	5.8% (4.2–8.0)	**< 0.001**	**3.97** **(3.15–5.00)**	**< 0.001**	1.09 (0.72–1.65)	0.68	**< 0.001**
Sexual violence disclosure^e^	74.0% (67.2–81.6)	69.1% (64.6–73.8)	0.25	84.7% (78.3–91.7)	75.4% (70.2–81.0)	**0.03**	**1.14** **(1.01–1.28)**	**0.03**	**1.09** **(1.00–1.19)**	**0.04**	0.58
Secondary School											
Past year sexual violence prevalence	22.7% (17.5–29.4)	20.1% (13.3–30.3)	0.62	15.1% (11.6–19.8)	23.1% (16.5–32.4)	**0.06**	**0.70** **(0.54–0.91)**	**0.01**	1.13 (0.78–1.67)	0.51	**0.04**
School incident rate^d^	*(33.9)* *(24.7–46.6)*	*(28.8)* *(17.7–46.9)*	0.59	*(26.2)* *(18.8–36.5)*	*(35.8)* *(25.4–50.3)*	0.20	0.82 (0.56–1.19)	0.29	1.23 (0.78–1.94)	0.38	0.18
High confidence	54.8% (43.1–69.7)	36.9% (30.6–44.5)	**0.01**	69.9% (63.5–77.0)	46.0% (36.6–57.8)	**0.001**	**1.30** **(1.01–1.66)**	**0.04**	1.24 (0.96–1.61)	0.10	0.82
Correct knowledge	26.4% (19.8–35.3)	13.5% (9.2–19.8)	**0.01**	67.8 (61.9–74.3)	18.9% (13.7–26.0)	**< 0.001**	**2.57** **(1.91–3.45)**	**< 0.001**	1.40 (0.93–2.09)	0.11	**0.02**
Sexual violence disclosure^e^	71.1% (61.7–81.9)	65.9% (54.5–79.8)	0.54	81.7% (72.7–91.8)	73.9% (67.2–81.3)	0.19	1.13 (0.93–1.38)	0.22	1.12 (0.92–1.36)	0.25	0.94

^a^Between-arm difference within time point; ^b^ Between-time point within arm ratio; ^c^ Between-arm between-time point ratio of ratios; ^d^ Per 1000 person-months; ^e^Among those affected (past year); boldface illustrates statistical significance at *p* < 0.05

### Secondary outcomes

Substantial differences in baseline self-defense confidence were seen across intervention and control groups (full confidence 41.2% vs. 26.2%, *p* < 0.001). Modest increases in confidence were observed for both intervention (RR = 1.33 [1.17–1.50]) and control (RR = 1.26[1.06–1.49]) groups; the interaction effect was not significant (*p* = 0.63). While baseline self-defense knowledge was slightly higher for the intervention than control group (full knowledge 15.0% vs. 7.0%, *p* < 0.001), knowledge increased three-fold in the intervention group by follow-up(RR_intervention_ = 3.33[2.76–4.02]), with no significant difference was observed in the control arm (RR_control_ = 1.27[0.95–1.70]; interaction effect *p*-value < 0.001.Among those who experienced sexual violence in the past year, violence disclosure increased in both intervention and control groups (Intervention 73.0% vs 83.7%; RR_Intervention_ = 1.14 [1.04–1.26], *p* = 0.007; Control 68.2% vs. 74.8%; RR_Control_ = 1.10 [1.01–1.19], *p* = 0.02; resulting in an insignificant interaction effect (*p* = 0.50).

### Stratification by primary vs. secondary school and district

In primary schools, past-year sexual violence prevalence decreased significantly in the intervention arm (baseline 13.0% vs. follow-up 7.5%; RR_Intervention_ 0.58 [95% CI 0.45–0.74]), and remained steady in the control arm (12.1% vs. 11.8%; RR_Control_ 0.97 [95% CI 0.78–1.20]); intervention effect *p*-value = 0.002. In primary schools, differences in sexual violence incident rates were not statistically significant, but in the direction of a decrease in the intervention arm and an increase in the control arm (Rate Ratio_Intervention_ = 0.88[0.71–1.11]; Rate Ratio_Control_ = 1.20[0.88–1.62]; overall interaction effect *p*-value = 0.12. Significant interaction effects were observed for self-defense knowledge (*p* < 0.001), while confidence and disclosure increased for both intervention and control arms with nonsignificant intervention interaction effects.

In secondary schools, where past-year sexual violence was most prevalent at baseline (22.7% intervention, 20.1% control), prevalence similarly decreased significantly in the intervention arm (RR_intervention_ = 0.70[0.54–0.91]) and remained unchanged in the control arm (RR_control_ = 1.13[0.78–1.67]); intervention effect p-value = 0.04). Differences in sexual violence incident rates were not statistically significant, though trended towards a reduction in intervention schools and an increase in control schools (Rate Ratio_Intervention_ = 0.82[0.56–1.19]; Rate Ratio_Control_ = 1.23[0.78–1.94]; overall interaction effect p-value = 0.18. Similar to primary schools, an interaction effect was observed for self-defense knowledge (*p* = 0.02), whereas confidence and disclosure increased for both arms with nonsignificant intervention interaction effects.

In district-stratified analysis (data not shown), significant interaction effects (*p* < 0.05) were observed for sexual violence prevalence in Lilongwe and Salima districts. In Dedza, intervention impact was concentrated among secondary students (interaction effect *p* = 0.03). The lack of significant effect observed for primary students in Dedza may reflect statistical power issues stemming from the low baseline prevalence of sexual violence among Dedza primary students (8.3% intervention arm; 11.5% control arm), coupled with their somewhat smaller overall sample size.

### Perpetrator mix

Overall, across intervention arms, time periods, and school levels, most sexual violence perpetrators were known to victims (Table 3). Boyfriends were the most common perpetrators for primary (34.5–45.4%) and secondary students (41.0–68.6%) across both intervention and control groups, followed by relatives, friends and neighbors. Only 9–15% of primary students affected by violence indicated strangers as perpetrators; < 1–9.5% among secondary students. Amongst the intervention group, the proportion of sexual assaults perpetrated by relatives decreased significantly from 21.0% at baseline to 9.8% at follow-up (*p* = 0.01). Perpetration by boyfriends, other known adults, and strangers held steady. Several changes in the control group were also noted during this time, including decreases in the proportion of assaults perpetrated by neighbors among those victimized. When stratified by school level, the decrease in relative-perpetrated violence was found concentrated among primary school students in the intervention arm (26.0 to 8.9%, *p* = 0.001). Among secondary school intervention arm students, there were no significant changes in sexual violence perpetrators, however in the control arm, a higher proportion of relatives and strangers were indicated as violence perpetrators at follow-up, while the proportion of boyfriends as perpetrators decreased from baseline to follow-up (68.6 to 41.0%; *p* = 0.004).

**Table 3:** Perpetrators* of past-year forced sex among those who experienced sexual violence at baseline and follow-up, respectively, by intervention status

	Intervention			Control		
	Baseline (*n* = 452) n (%)	Follow-Up (*n* = 194) n (%)	*p*-value^a^	Baseline (*n* = 423) n (%)	Follow-Up (*n* = 268) n (%)	*p*-value^a^
All Students						
Boyfriend	203 (44.9)	90 (46.4)	0.46	188 (44.4)	117 (43.7)	0.75
Friend	51 (11.3)	30 (15.5)	0.10	41 (9.7)	33 (12.3)	0.33
Relative	95 (21.0)	19 (9.8)	**0.01**	79 (18.7)	57 (21.3)	0.47
Neighbor	37 (8.2)	23 (11.9)	0.15	46 (10.9)	13 (4.9)	**0.01**
Known Adult	24 (5.3)	10 (5.2)	0.95	29 (6.9)	16 (6.0)	0.66
Stranger	32 (7.1)	21 (10.8)	0.10	28 (6.6)	28 (10.5)	0.19
Other	10 (2.2)	**	0.19	12(2.8)	**	0.21
Primary School Only						
Boyfriend	103 (35.6)	45 (36.6)	0.57	103 (34.5)	74 (45.4)	**0.05**
Friend	32 (11.1)	24 (19.5)	**0.02**	32 (10.7)	26 (16.0)	0.15
Relative	75 (26.0)	11 (8.9)	**0.001**	62 (20.7)	26 (16.0)	0.08
Neighbor	25 (8.7)	18 (14.6)	0.08	39 (13.0)	5 (3.1)	**< 0.001**
Known Adult	20 (6.9)	6 (4.9)	0.59	25 (8.4)	12 (7.4)	0.68
Stranger	29 (10.0)	18 (14.6)	0.14	27 (9.0)	18 (11.0)	0.63
Other	**	**	0.53	11 (3.7)	**	0.12
Secondary School Only						
Boyfriend	100 (61.4)	45 (63.4)	0.61	85 (68.6)	43 (41.0)	**0.004**
Friend	19 (11.7)	6 (8.5)	0.54	9 (7.3)	7 (6.7)	0.90
Relative	20 (12.3)	8 (11.3)	0.95	17 (13.7)	31 (29.5)	**0.001**
Neighbor	12 (7.4)	5 (7.0)	0.99	7 (5.7)	8 (7.6)	0.42
Known Adult	4 (2.5)	4 (5.6)	0.14	4 (3.2)	4 (3.8)	0.82
Stranger	**	3 (4.2)	0.37	**	10 (9.5)	**0.03**
Other	5 (3.1)	**	**< 0.001**	**	**	0.32

boldface illustrates statistical significance at *p* < 0.05

*not mutually exclusive

^**^cell with < 2% suppressed

^a^generalized linear models with binomial distribution and logit link and school-correlated variance

### Skills mix

Within the intervention group, 43% of girls said that they had used skills to stop forced sex since IMPower ESD training. Of the girls that used the skills, 49% used verbal only, 13% used physical only, and 38% used a combination of verbal and physical skills. Of those, 52% reported using the learned skills more than once to stop forced sex. The skills learned within IMPower extended beyond sexual violence prevention, with an additional 53% of girls reporting using the skills to stop harassment and 52% reporting using the skills to stop physical violence since the ESD training (data not shown).

### Disclosure

Those who disclosed sexual violence were most likely to turn to friends (approximately 50% at both baseline and follow up), with fewer than 7% reporting to law enforcement (data not shown). While approximately 80% of girls disclosed their violence experience to someone, they chose to disclose to friends and other members of their informal networks, instead of police or medical professionals.

## Discussion

Results from this cluster-randomized controlled implementation trial support the effectiveness of school-based, empowerment self-defense training as a promising strategy in reducing risk for sexual violence for girls in both primary and secondary schools in Malawi. Compared with control participants, students assigned to IMPower intervention reported reductions in past-year sexual violence from baseline to follow-up, and increases in self-defense-related knowledge. IMpower has been previously found effective in reducing sexual violence in the densely-populated urban settlements of Nairobi, Kenya [27, 28]; current findings provide a critical replication and extension of this work by demonstrating similar reductions in sexual assault prevalence, as well as increases in knowledge in self-defense, in the far more diffuse setting of Malawi (197.5 people/km_sq_; 2017) [33],across a heterogeneity of districts, and across both primary and secondary school levels. Results provide timely direction for addressing the epidemic of sexual violence against young women in Malawi, where an estimated one in four young women experience sexual violence by the time of secondary school.

Several potential mechanisms may underlie the reductions observed in sexual violence among ESD participation in this study, and in past evaluations [27, 28, 32]. The observed increases in self-defense knowledge in the intervention arm relative to controls, may in part explain the reductions in sexual violence. Empowerment and social cognitive theory would suggest knowledge as necessary but insufficient to change in health-related behavior, and would  suggest power, self-efficacy, or confidence as necessary to implement change. Surprisingly, confidence in responding to threatening situations increased in both intervention and control arms with no significant intervention effect observed. It is possible that our current measures, while tailored to IMpower content, were insufficiently sensitive to capture meaningful changes in confidence and empowerment resulting from the program. It is also possible that natural maturation is responsible for the observed increases in confidence across both arms. Our findings should be interpreted with caution given measurement limitations in assessing self-confidence (2 items), particularly given recent evidence from Nairobi, which showed increases in self-efficacy as assessed on a generalized scale, when IMpower was implemented in concert with programming for young men [32]. Finally, it is possible that IMpower shifted participants’ interpersonal power in ways that were perceived by potential sexual violence perpetrators and changed their behavior. Evaluating this mechanism would require community-level research, in addition to the participant data that has served as the basis for this and related studies.

The vast majority of sexual violence perpetrators were known to victims, with boyfriends the most commonly reported perpetrators at baseline and follow-up, across primary and secondary schools and intervention and control groups. Results are consistent with past research in Malawi and globally indicating boyfriends, intimate partners and other acquaintances as leading perpetrators of violence against women [15, 34]. In the control arm, among secondary students, proportion of violence survivors reporting boyfriends as perpetrators decreased while proportion of survivors reporting relatives as perpetrators increased by follow-up. Intervention arm primary schools saw decreases in the proportion of survivor reporting relatives as perpetrators by follow-up. Further research, including qualitative inquiry, can help inform the ways in which this intervention may function differentially for different types of perpetrators. Differences observed in perpetrator mix at baseline and follow-up, however results should be interpreted with caution in that they represent the portion of survivors reporting a given perpetrator type rather than prevalence or incidents by perpetrator.

Results should be considered in light of several additional limitations. As in past evaluations of this program [27, 28, 32], the inability to link individual surveys across baseline and follow-up constrains evaluation of mechanisms responsible for changes observed, and discernment of primary prevention (preventing new incidents among unaffected individuals) vs secondary prevention (reducing recurrent exposure among those who have experienced sexual violence). The evaluation’s emphasis on efficient survey data collection for this demonstration project prompted some measurement limitations. Most notably, we lack data on demographic characteristics that could be used to explore effects by specific groupings. Sexual violence assessment was limited to forced sex, which offers specificity yet overlooks non-consensual sexual experiences resulting from coercion, threats or pressure that did not rise to the level of physical force, and thus may underestimate the extent of sexual violence. Future research should also explore intervention effects on sexual harassment and  physical and emotional abuse, which were not assessed for time considerations. The relatively short follow-up duration limits our ability to understand the durability of findings. The self-reported nature of data, single-blind design with students, but not school or study staff, blind to intervention assignment, and instructor-led survey administration could introduce error or bias, despite the measures in place to enhance confidentiality and comfort. Our use of a past-year prevalence outcome for sexual violence is a limitation in precision for follow-up estimates given the 10.5 month length to follow-up; this imprecision is evenly distributed across intervention and control arms and is not likely to introduce bias. Statistical inferences were consistent in sensitivity analysis converted to adjust for time difference. While the IMpower program targets girls specifically, we note that sexual violence also affects young men [15]. Finally we note that while IMPower is designed to increase skills, confidence, and safety behavior in responding to sexual violence and potentially threatening situations, the ultimate behavioral responsibility for sexual violence prevention rests with perpetrators, not victims, of violence.

## Conclusions

Our study adds to a limited evidence base on sexual violence prevention interventions that are effective across a diversity of settings, and address the needs of younger girls in particular. It is worth noting that this intervention has now been piloted and evaluated in two African countries, Kenya and Malawi, demonstrating efficacy across a diversity of cultural and social systems.

Results support the effectiveness of ESD to address sexual violence in this high prevalence setting. ESD may aid Malawi and other signatory nations in meeting the obligations set forth by the Convention on the Elimination of All Forms of Discrimination Against Women (CEDAW), and approach the goal of elimination of violence against women and girls set forth with Sustainable Development Goal #5. Implementation within the education system can enable sustainability and reach.

## Abbreviations

CEDAW: Convention on the Elimination of All Forms of Discrimination Against Women
CI: Confidence interval
ESD: Empowerment self-defense
GBV: Gender-based violence
IRB: Institutional review board
LMIC: Low and middle-income country
NMNW: No means no worldwide
RR: Risk ratio
SD: Standard deviation
